# Agreement between EMS provider-assigned prehospital triage and initial emergency department triage in pediatric and adult EMS-transported encounters: A retrospective observational study

**DOI:** 10.1371/journal.pone.0352969

**Published:** 2026-07-06

**Authors:** Min-Jung Kim, Hwan Sun Moon, So-Hyun Paek

**Affiliations:** 1 Department of Emergency Medicine, CHA Bundang Medical Center, CHA University, Seongnam, Republic of Korea; 2 Central 119 Rescue Headquarters, National Fire Agency, Sejong, Republic of Korea; 3 Department of Medical Sciences, Graduate School of Ajou University, Suwon, Republic of Korea; University of Hafr Al-Batin, SAUDI ARABIA

## Abstract

**Background:**

Prehospital triage supports destination decisions, resource activation, and early risk stratification, yet accurate triage of children in the field remains challenging. In South Korea, a five-level emergency department triage framework has recently been extended to the Prehospital setting through nationwide implementation of a prehospital triage scale. This study aimed to evaluate agreement between EMS provider-assigned prehospital triage and initial ED triage, with a pediatric-focused primary analysis and adults as a secondary comparator.

**Methods:**

We conducted a retrospective observational study using linked EMS run-sheet and emergency department records from one university hospital during the first year of nationwide implementation (February–December 2024). We included all EMS-transported emergency department encounters that were registered and assigned an initial emergency department triage level on arrival. Agreement between prehospital and emergency department triage levels was assessed using overall exact agreement, Cohen’s unweighted κ, and quadratically weighted κ.

**Results:**

Among 5,182 EMS-transported emergency department encounters during the study period, 4,729 were included in the final analysis, comprising 1,242 pediatric and 3,487 adult encounters. Overall agreement was 47.7% in pediatric encounters and 52.8% in adults. Quadratically weighted κ indicated modest agreement in children (0.27) and higher agreement in adult encounters (0.40). Disagreement was concentrated at adjacent-level boundaries, particularly between levels 2–3 and 3–4. Discordance toward higher prehospital acuity predominated in pediatric encounters, whereas discordance toward lower prehospital acuity was more common in adults.

**Conclusions:**

During the first year of nationwide implementation, prehospital and emergency department triage showed modest agreement, with lower concordance and a predominance of discordance toward higher prehospital acuity in pediatric encounters. Adjacent-level boundaries represent practical targets for pediatric-focused training and feedback to improve consistency of prehospital triage assignments.

## Introduction

Triage is a core process in emergency care and prehospital systems, intended to match patient urgency to available resources and to support timely interventions [1,2]. Agreement and reliability of triage scales vary by setting and rater training, and pediatric triage is consistently more challenging than adult triage because children have age-dependent vital sign ranges, less specific clinical presentations, and communication limitations that can increase uncertainty during acuity assignment [2–5].

In South Korea, the Korean Triage and Acuity Scale (KTAS) is a five-level triage system that has been used in emergency departments (EDs) since 2016 [6,7]. ED-based studies have reported that KTAS is generally reliable and clinically informative but remains susceptible to mistriage, particularly near adjacent-level boundaries and when modifiers are applied inconsistently [8–11].

In the prehospital setting, Korean emergency medical services (EMS) historically used a four-level priority system (P1–P4) that depended heavily on provider experience and did not fully mirror the ED triage framework. To harmonize triage across the prehospital–ED continuum, the Korean Society of Emergency Medicine KTAS Committee developed the Prehospital Korean Triage and Acuity Scale (Pre-KTAS), which the National Fire Agency implemented nationwide beginning in February 2024 [12–14]. In this study, Pre-KTAS refers to an acuity level assigned by EMS providers during the prehospital encounter after patient contact, rather than a dispatcher-assigned priority code. Despite nationwide implementation, evidence on the real-world field performance of Pre-KTAS remains limited, particularly among pediatric patients transported by EMS [15–18].

Internationally, the use of a shared multi-level triage framework across both prehospital and ED settings is not universal, and pediatric evidence on cross-setting agreement remains limited [19,20]. Initial ED KTAS was used as a pragmatic comparator because it is the routinely documented ED triage level assigned by trained triage personnel within the same national five-level triage framework; it was not considered a definitive gold standard.

We aimed to evaluate encounter-level agreement between EMS provider-assigned prehospital triage and initial ED triage within the KTAS-aligned five-level framework, with pediatric EMS-transported encounters as the primary analytic focus and adult encounters as a secondary comparator.

## Materials and methods

### Study design and aim

The primary aim of this retrospective observational study was to quantify encounter-level agreement between EMS provider-assigned prehospital triage and initial ED triage within a KTAS-aligned five-level triage framework, with pediatric EMS-transported ED encounters as the primary analytic focus and adult encounters as a secondary comparator. To address this aim, we used routinely collected EMS run-sheet and ED electronic medical record data that were deterministically linked at the encounter level [21,22].

### Study setting and data sources

The study was conducted at Bundang CHA Medical Center (CHA University), a university-affiliated hospital in Seongnam, Republic of Korea. The study combined two routinely collected data sources: (1) EMS run-sheet data from the National Fire Agency and (2) ED electronic medical record (EMR) data from Bundang CHA Medical Center. The study period was 1 February 2024 to 31 December 2024.

### Study population

We included all EMS-transported ED encounters at the study hospital between 1 February and 31 December 2024 that were registered and assigned an initial ED KTAS level at ED registration. We excluded encounters with missing prehospital Pre-KTAS or no completed ED registration after EMS transport. Pediatric encounters were defined as age < 15 years at ED arrival, and adult encounters as age ≥ 15 years according to the KTAS age threshold [23].

### Triage framework and comparison

Pre-KTAS is a five-level prehospital acuity assessment recorded by EMS providers during the prehospital encounter and was implemented nationwide beginning in February 2024 [14]. In this study, Pre-KTAS refers to the acuity level assigned by EMS providers after patient contact and before ED registration; it was not a dispatcher-assigned priority code. The EMS dataset did not contain a precise timestamp indicating the exact moment of Pre-KTAS assignment within the EMS mission. Therefore, Pre-KTAS was analyzed as an encounter-level prehospital triage assessment.

The ED comparator for this study was the initial KTAS level assigned at ED registration by trained triage personnel according to national KTAS guidance [23]. Initial ED KTAS was used as a pragmatic comparator because it is routinely documented within the same national five-level triage framework; it was not considered a definitive gold standard for triage correctness. Pre-KTAS and KTAS are ordinal five-level scales in which level 1 indicates the highest acuity. KTAS applies separate pediatric and adult triage criteria using a 15-year age threshold; therefore, pediatric and adult analyses were prespecified accordingly [23]. The core comparison in this study was encounter-level agreement between EMS provider-assigned Pre-KTAS and initial ED KTAS.

### Record linkage, data governance, and ethics

EMS run-sheet data and ED electronic medical record data were deterministically linked at the encounter level by the data custodians using the EMS run-sheet serial number and ED encounter registration records. Linkage quality was checked using sex, age, EMS transport timestamps, and ED registration timestamps to ensure a unique one-to-one match.

Encounters were excluded from the analytic cohort if they had missing prehospital Pre-KTAS or no completed ED registration after EMS transport. Because linkage was performed by the data custodians before release of the de-identified analytic dataset, a separate linkage success rate could not be independently recalculated by the investigators after receipt of the dataset.

The final analytic dataset contained no direct personal identifiers. The de-identified linked dataset was accessed for research purposes on 1 September 2025. The authors did not have access to information that could identify individual participants during or after data collection. The study was approved by the Institutional Review Board of Bundang CHA Medical Center (CHAMC 2025-07-027), with a waiver of informed consent.

### Variables

Baseline variables included age and sex. Time-related variables included month/season and weekday versus weekend presentation. Clinical characteristics included visit type (disease vs non-disease) and EMS-recorded chief complaint categories, where available. The non-disease category included injury-related or external-cause presentations, including trauma, burns, and poisoning, as recorded in the source data. Chief complaint categories were derived from EMS run-sheet free-text fields using keyword-based categorization. Missing, ambiguous, or non-classifiable complaints were retained as an unclassified or other category rather than excluded from the relevant exploratory analyses. Comorbidity status was derived from the EMR and categorized as yes/no/unknown based on documentation of chronic conditions.

ED course variables included disposition (discharge, general ward admission, intensive care unit admission, inter-hospital transfer, or death in the ED) and ED length of stay, defined as the time from ED registration to ED departure. ED length of stay was categorized as <1 h (0–59 min), 1–2 h (60–119 min), 2–4 h (120–239 min), 4–6 h (240–359 min), 6–12 h (360–719 min), or ≥12 h (≥720 min).

### Outcomes

The primary outcome was agreement between EMS provider-assigned Pre-KTAS and initial ED KTAS, assessed using: (1) overall exact agreement (%), (2) Cohen’s kappa (unweighted), and (3) quadratically weighted kappa with 95% confidence intervals [24,25]. Quadratic weights were applied to reflect the ordinal structure of the five-level triage scale, assigning increasing penalties to disagreements further from the diagonal. Ninety-five percent confidence intervals were estimated using asymptotic standard errors.

Secondary outcomes described the direction and magnitude of discordance between EMS provider-assigned Pre-KTAS and initial ED KTAS. Discordance toward higher prehospital acuity was defined as a lower numeric level in Pre-KTAS than in ED KTAS, whereas discordance toward lower prehospital acuity was defined as a higher numeric level in Pre-KTAS than in ED KTAS. These terms describe the direction of discordance only and do not imply clinical correctness or superiority of either triage assignment. Discordance was summarized as exact match, ± 1 level, and ≥±2 levels, and we described boundaries with concentrated discordance, such as 2–3 and 3–4.

### Data and statistical analysis

Continuous variables were summarized as mean (standard deviation) or median (interquartile range), depending on distribution, and categorical variables as counts (%). Pediatric and adult cohorts were compared using chi-square tests (or Fisher’s exact tests when appropriate) for categorical variables and independent-samples t tests or Wilcoxon rank-sum tests for continuous variables.

Agreement statistics were estimated overall and stratified by age group. Prespecified subgroup analyses were conducted by visit type (disease vs non-disease) and major chief complaint categories, where available. Missing data for the primary outcome variables (Pre-KTAS or ED KTAS) were handled by exclusion. For covariates, missingness was retained using an “unknown” or “unclassified” category when applicable.

Because this retrospective study included all eligible encounters during the study period and was designed as a descriptive agreement study rather than a causal inference study, no formal a priori sample size calculation was performed. All analyses were conducted using SAS version 9.4 (SAS Institute Inc., Cary, NC, USA). Two-sided p values <0.05 were considered statistically significant.

## Results

### Study population and excluded encounters

**Fig 1:**
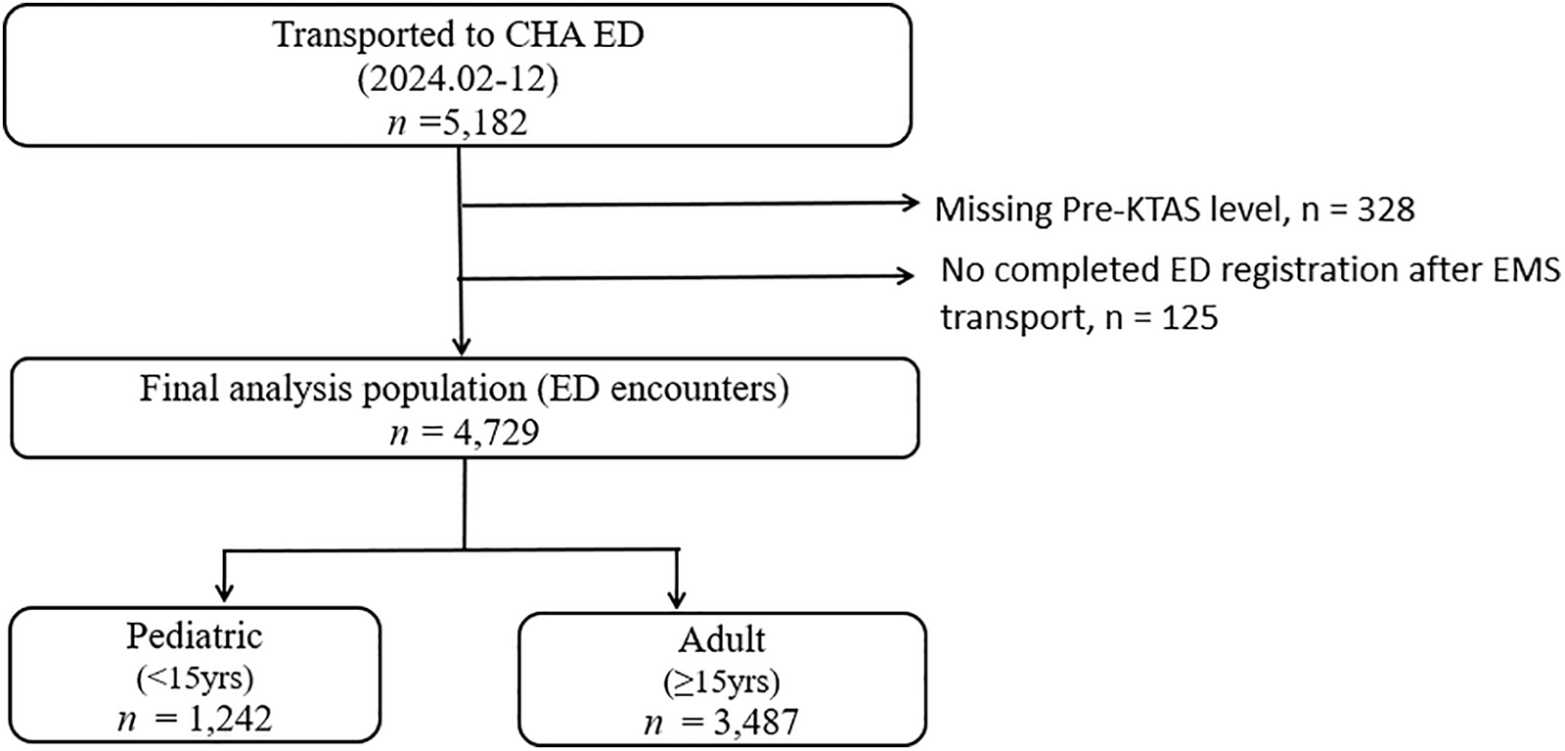
Flow of EMS-transported encounters included in the analysis. Among encounters transported by EMS to the Emergency Department of Bundang CHA Medical Center between February and December 2024 (n = 5,182), encounters were excluded if the prehospital Pre-KTAS level was missing (n = 328) or if ED registration was not completed after EMS transport (n = 125). The final analytic cohort consisted of 4,729 ED encounters, stratified into pediatric (<15 years; n = 1,242) and adult (≥15 years; n = 3,487) groups according to the KTAS age threshold. Numbers represent encounters.

A total of 5,182 EMS-transported encounters to the study ED were identified during the study period. After excluding 328 encounters with missing prehospital Pre-KTAS and 125 encounters with no completed ED registration after EMS transport, the final analytic cohort included 4,729 EMS-transported ED encounters, comprising 1,242 pediatric encounters (<15 years) and 3,487 adult encounters (≥15 years) (**Fig 1**).

Baseline characteristics of included and excluded encounters are presented in **S1 Table**. Among encounters excluded for missing Pre-KTAS, the proportion of non-disease encounters was higher than among included encounters (20.7% vs 12.3%, P < 0.001), whereas the proportion of pediatric encounters was lower (15.1% vs 26.3%, P < 0.001). Among the 125 encounters with no completed ED registration after EMS transport, 114 had available EMS data for comparison; these encounters showed a higher proportion of non-disease encounters (P < 0.001) and younger median age (44.5 vs 60 years, P = 0.011). The remaining 11 encounters could not be compared because linked data were unavailable.

### Baseline characteristics

Baseline characteristics of pediatric and adult EMS-transported ED encounters are summarized in **Table 1**. Pediatric encounters more frequently involved non-disease presentations and had fewer documented comorbidities than adult encounters. Although several baseline characteristics differed statistically between age groups, most effect size estimates were small based on Cramér’s V (**Table 1**).

**Table 1:** Baseline characteristics of pediatric and adult ED encounters.

	All		Pediatric (<15)		Adult (>=15)		P Value	Cramér’s V
	N	%	N	%	N	%		
**Total**	4729	100.0	1242	100.0	3487	100.0		
**Age**								
Median (IQR)	60(12-75)		2(1-5)		68(55-79)			
**Gender**							0.030	0.032
Male	2659	56.2	731	58.9	1928	55.3		
**Season**							< 0.001	0.065
Spring	1158	24.5	284	22.9	874	25.1		
Summer	1300	27.5	402	32.4	898	25.8		
Autumn	1179	24.9	291	23.4	888	25.5		
Winter	1092	23.1	265	21.3	827	23.7		
**Visit type**							< 0.001	0.108
Disease	4104	86.8	1016	81.8	3088	88.6		
Non-disease	580	12.3	222	17.9	358	10.3		
Others	45	1.0	4	0.3	41	1.2		
**Weekend**							0.330	0.014
Weekday	3386	71.6	876	70.5	2510	72.0		
Weekend	1343	28.4	366	29.5	977	28.0		
**ED Disposition**							<0.001	0.284
Admitted	1933	40.9	243	19.6	1690	48.5		
Discharged	2616	55.3	974	78.4	1642	47.1		
Transfer-out	51	1.1	18	1.4	33	0.9		
Death	129	2.7	7	0.6	122	3.5		
**ED Duration**								
< 1 h *(0–59 min)*	326	6.9	280	22.5	46	1.3	<0.001	0.468
1– < 2 h *(60–119 min)*	614	13.0	258	20.8	356	10.2		
2– < 4 h *(120–239 min)*	1764	37.3	538	43.3	1226	35.2		
4– < 6 h *(240–359 min)*	1114	23.6	107	8.6	1007	28.9		
6– < 12 h *(360–719 min)*	749	15.8	46	3.7	703	20.2		
≥ 12 h *(≥720 min)*	153	3.2	12	1.0	141	4.0		
Unknown	9	0.2	1	0.1	8	0.2		
**Comorbidity**							<0.001	0.649
Yes	3214	68.0	238	19.2	2976	85.3		
No	1425	30.1	994	80.0	431	12.4		
Unknown	90	1.9	10	0.8	80	2.3		

Values are n (%) unless otherwise indicated. Age is presented as median (IQR) without between-group hypothesis testing. ED length of stay was defined as time from ED registration to ED departure. Unknown indicates missing or invalid entries. P values are presented for completeness; however, given the large sample size, emphasis is placed on effect size estimates (Cramér’s V) to assess the magnitude of differences. Cramér’s V values were interpreted as negligible (<0.1), small (0.1–0.3), moderate (0.3–0.5), or large (>0.5).

### Agreement between prehospital pre-KTAS and initial ED KTAS

**Fig 2:**
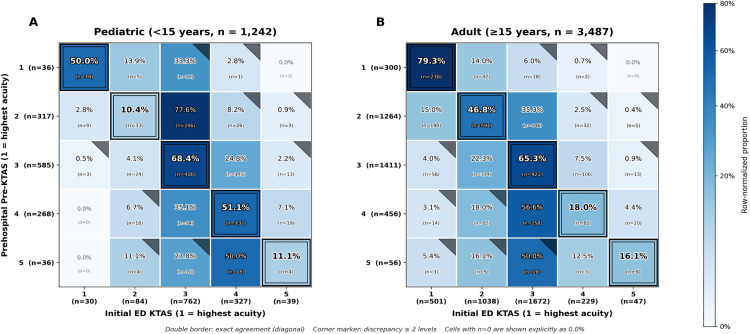
Row-normalized heatmaps of pre-KTAS versus initial ED KTAS. Panel A shows pediatric encounters (<15 years) and Panel B shows adult encounters (≥15 years). Rows indicate Pre-KTAS levels assigned by EMS and columns indicate initial ED KTAS levels assigned at ED registration (1 = highest acuity). Each cell displays the row-normalized proportion (%) and the number of encounters (n), with color intensity reflecting the row-normalized proportion on a sequential blue scale. Diagonal cells (exact agreement) are highlighted with double borders. Corner markers indicate discrepancies of ≥2 triage levels. Cells with zero encounters are shown explicitly as 0.0%. Column totals are shown in parentheses on the x-axis tick labels. The underlying 5 × 5 contingency tables (counts only) are provided in S2 Table.

Heatmaps of prehospital Pre-KTAS versus initial ED KTAS are shown in **Fig 2**, and the underlying 5 × 5 contingency tables are provided in **S2 Table**. Overall exact agreement was 47.7% in pediatric encounters and 52.8% in adult encounters.

Agreement statistics are presented in **Table 2**: the unweighted κ was 0.18 in pediatric encounters and 0.30 in adult encounters, and the quadratically weighted κ was 0.27 and 0.40, respectively. Sensitivity analyses using Gwet’s AC1 showed higher agreement estimates than unweighted κ in both age groups, but the relative pattern of lower agreement in pediatric encounters remained unchanged (**S3 Table**).

**Table 2:** Agreement between prehospital pre-KTAS and initial ED KTAS by age group.

Group	Encounters (n)	Unweighted κ (95% CI)	Quadratically weighted κ (95% CI)
Pediatric (<15 years)	1242	0.18 (0.14–0.22)	0.27 (0.23–0.31)
Adults (≥15 years)	3487	0.30 (0.28–0.33)	0.40 (0.38–0.43)

Pre-KTAS indicates the Prehospital Korean Triage and Acuity Scale assigned by EMS, and initial ED KTAS indicates the Korean Triage and Acuity Scale level assigned at ED registration (1 = highest acuity). Unweighted κ was calculated using Cohen’s kappa. Quadratically weighted κ was calculated to account for the ordinal 5-level triage scale, applying quadratic weights to disagreements between categories. Ninety-five percent confidence intervals (95% CI) were estimated using asymptotic standard error (normal approximation). Pediatric encounters were defined as <15 years and adult encounters as ≥15 years according to the KTAS age threshold.

Exploratory analyses stratified by pediatric age subgroup showed that agreement was lowest among children aged 1–4 years (overall agreement 46.0%; unweighted κ, 0.12; quadratically weighted κ, 0.23), whereas infants aged <1 year showed the highest agreement (overall agreement 50.9%; unweighted κ, 0.29; quadratically weighted κ, 0.41) (**S4 Table**). Across all pediatric age subgroups, discordance toward higher prehospital acuity was more frequent than discordance toward lower prehospital acuity.

### Direction and magnitude of discordance

**Fig 3:**
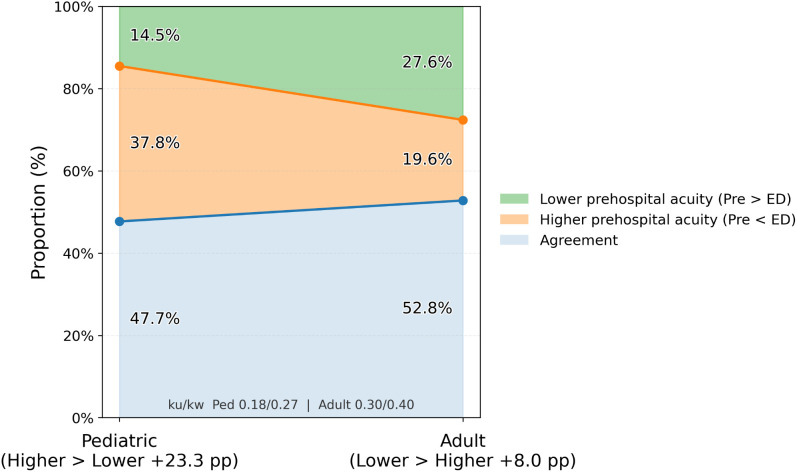
Direction of discordance between prehospital Pre-KTAS and initial ED KTAS by age group. Horizontal diverging bar plot showing, for pediatric (<15 years) and adult (≥15 years) encounters, the proportions of discordance toward lower prehospital acuity (left) and discordance toward higher prehospital acuity (right) between prehospital Pre-KTAS and initial ED KTAS. Discordance toward lower prehospital acuity was defined as a higher numeric Pre-KTAS level than ED KTAS, whereas discordance toward higher prehospital acuity was defined as a lower numeric Pre-KTAS level than ED KTAS. Agreement is shown as an annotation at the midline (100% minus the two discordant proportions). Unweighted kappa and quadratically weighted kappa are annotated for each age group.

Disagreement was concentrated at adjacent triage levels, particularly at the 2–3 and 3–4 boundaries, whereas discrepancies of two or more triage levels were uncommon (pediatric: 7.2% [90/1,242]; adults: 7.5% [262/3,487]). Within each prehospital triage level, exact agreement with initial ED KTAS was highest at Pre-KTAS level 3 in pediatric encounters (68.4%, 400/585) and at Pre-KTAS level 1 in adult encounters (79.3%, 238/300), whereas it was lowest at Pre-KTAS level 2 in pediatric encounters (10.4%, 33/317) and at Pre-KTAS level 5 in adult encounters (16.1%, 9/56) (**Fig 2**). The direction of discordance is summarized in **Fig 3**. In pediatric encounters, discordance toward higher prehospital acuity was more frequent than discordance toward lower prehospital acuity (37.8% vs 14.5%), whereas in adult encounters discordance toward lower prehospital acuity was more frequent than discordance toward higher prehospital acuity (27.6% vs 19.6%).

In exploratory analyses stratified by visit type, discordance direction differed between disease and non-disease encounters within both age groups (**S5 Table**). Among pediatric encounters, disease encounters showed more frequent discordance toward higher prehospital acuity than non-disease encounters (39.3% vs 31.5%). Among adult encounters, non-disease encounters had lower exact agreement (48.9% vs 53.3%) but higher weighted κ than disease encounters (0.48 vs 0.38).

In exploratory analyses restricted to pediatric encounters assigned prehospital Pre-KTAS level 2, seizure/convulsion (59.0%) and fever (19.6%) were the most frequent chief complaint categories. Across most categories, discordance toward higher prehospital acuity predominated, whereas exact agreement remained low overall (10.4%) (**S6 Table**).

### ED disposition according to discordance direction

To describe clinical disposition patterns according to discordance direction, ED disposition was compared across three groups: discordance toward higher prehospital acuity, exact agreement, and discordance toward lower prehospital acuity (**Table 3**). In adult encounters, admission or death was more frequent in the exact agreement and discordance toward lower prehospital acuity groups than in the discordance toward higher prehospital acuity group. In pediatric encounters, differences in admission or death across discordance groups were less pronounced and did not reach statistical significance.

**Table 3:** Association between direction of discordance and ED disposition outcomes.

Panel A. Pediatric encounters (<15 years, n = 1,242)				
	Higher prehospital acuity (n = 470)	Agreement (n = 592)	Lower prehospital acuity (n = 180)	P Value
Admission, n (%)	84 (17.9)	112 (18.9)	47 (26.1)	0.052
Death, n (%)	0 (0.0)	7 (1.2)	0 (0.0)	0.021^†^
Admission or death, n (%)	84 (17.9)	119 (20.1)	47 (26.1)	0.064
Transfer-out, n (%)	4 (0.9)	12 (2.0)	2 (1.1)	0.259
Discharge, n (%)	382 (81.3)	461 (77.9)	131 (72.8)	0.056
**Panel B. Adult encounters (≥15 years, n = 3,487)**				
	**Higher prehospital acuity (n = 684)**	**Agreement (n = 1,842)**	**Lower prehospital acuity (n = 961)**	**P Value**
Admission, n (%)	242 (35.4)	929 (50.4)	519 (54.0)	< 0.001
Death, n (%)	0 (0.0)	107 (5.8)	15 (1.6)	< 0.001
Admission or death, n (%)	242 (35.4)	1,036 (56.2)	534 (55.6)	< 0.001
Transfer-out, n (%)	11 (1.6)	16 (0.9)	6 (0.6)	0.112
Discharge, n (%)	431 (63.0)	790 (42.9)	421 (43.8)	< 0.001

Discordance toward higher = Pre-KTAS level numerically lower than ED KTAS (prehospital classification more urgent). Discordance toward lower prehospital acuity = Pre-KTAS level numerically higher than ED KTAS (prehospital classification less urgent). P values from Pearson’s chi-square test comparing three discordance groups for each outcome. ^†^Fisher’s exact test used due to expected cell counts <5. Admission or death is a composite outcome. ED disposition categories (admission, death, transfer-out, discharge) are mutually exclusive; discharge includes all non-admission outcomes (symptom improvement, voluntary discharge, and outpatient follow-up).

Exploratory analyses of EMS crew composition, used as a proxy for provider training level, showed no consistent pattern of agreement by crew configuration (**S7 Table**).

## Discussion

### Main findings

In this linked EMS–hospital dataset from the first year of nationwide Pre-KTAS implementation, agreement between EMS provider-assigned prehospital Pre-KTAS and initial ED KTAS was modest, with lower concordance in pediatric encounters than in adult encounters. The direction of discordance also differed by age group: pediatric encounters more often showed discordance toward higher prehospital acuity, whereas adult encounters more often showed discordance toward lower prehospital acuity. In exploratory analyses by visit type, disease encounters in the pediatric cohort showed more frequent discordance toward higher prehospital acuity than non-disease encounters, suggesting that presentation type may contribute to cross-setting triage discordance. Importantly, discordance was concentrated at adjacent triage levels, particularly around 2–3 and 3–4 boundaries, whereas discrepancies of two or more triage levels were uncommon. These findings suggest that most disagreement reflected borderline triage decisions rather than extreme discordance, providing practical targets for focused education and feedback during early implementation.

### Comparison with previous literature

Consistent with prior ED-based KTAS studies, our findings suggest that disagreement is most likely to occur around borderline categories where chief complaint selection and modifier application influence triage assignment [8–11]. However, ED-based triage reliability studies are not directly equivalent to cross-setting agreement studies because the timing, environment, available information, and raters differ between EMS and ED settings. Our study extends this literature to the prehospital–ED continuum by directly comparing EMS provider-assigned Pre-KTAS with initial ED KTAS, which was used as a pragmatic comparator within a shared five-level triage framework rather than as a definitive gold standard.

Prehospital–ED discordance should therefore be interpreted as disagreement between two care settings rather than definitive misclassification by either system. EMS providers assign Pre-KTAS during the prehospital encounter with limited diagnostic information, evolving clinical conditions, and transport-related constraints. In contrast, initial ED KTAS is assigned after ED arrival and may be influenced by additional clinical information as well as operational factors such as crowding, staffing patterns, triage workload, and local workflow. These contextual differences may partly explain why adjacent-level discordance was common, whereas discrepancies of two or more triage levels were relatively uncommon.

From an international perspective, prehospital patient prioritization and ED triage have often relied on different instruments, and evidence on the use of a single triage framework across multiple points of the EMS–ED care continuum remains limited [19]. A systematic review noted a paucity of evidence regarding prehospital triage systems and the use of the same triage system across settings [19]. Nevertheless, shared or closely aligned triage frameworks have been implemented in some jurisdictions; for example, RETTS has been reported to be used across both prehospital and ED settings in parts of Scandinavia [19,26]. Similarly, in Geneva State, Switzerland, the Swiss Emergency Triage Scale, originally developed for ED triage, has been evaluated for use by paramedics in the prehospital setting, providing another example of extending a hospital-based triage framework to EMS care [27]. Studies evaluating triage agreement across prehospital and ED settings have generally reported only moderate agreement [17,27,28]. In this context, our findings provide early real-world evidence from a nationwide EMS implementation of a KTAS-aligned prehospital triage framework.

The lower agreement observed in pediatric encounters is also consistent with the broader pediatric triage literature. Prior pediatric ED studies have shown that agreement among triage providers can be limited, reflecting the inherent difficulty of assigning acuity in children [29]. Contemporary reviews further emphasize that pediatric triage remains challenging because of age-dependent physiology, non-specific presentations, communication barriers, and variation in how triage tools incorporate vital signs and modifiers [30]. More recent evidence also suggests that pediatric triage accuracy may vary by institutional context, including pediatric-specific versus general emergency departments [31]. Our study extends this literature by showing that lower pediatric concordance persists even when prehospital and ED triage are based on a shared five-level national framework. Pediatric-focused evidence specifically examining prehospital–ED agreement remains limited. Prior pediatric work has often involved different triage tools between the prehospital and ED stages, requiring subsequent re-mapping, and still reported lower agreement in children than adults [20]. By evaluating EMS provider-assigned Pre-KTAS and initial ED KTAS during the first year of nationwide Pre-KTAS implementation, our study provides an early benchmark for pediatric prehospital–ED triage agreement in routine practice.

### Pediatric-specific implications

Several factors may plausibly contribute to lower pediatric concordance. Pediatric triage is intrinsically challenging because normal vital sign ranges vary substantially with age, presentations are often non-specific, and communication barriers can limit symptom characterization, all of which may promote conservative higher-acuity decisions [5,30]. In addition, Pre-KTAS was implemented nationwide in 2024, and early implementation may have been accompanied by heterogeneous training exposure and learning curves across EMS providers, potentially amplifying variability around borderline levels [14,15].

The exploratory pediatric subgroup analyses support this interpretation. Age-stratified analyses suggested heterogeneity within the pediatric population, with the lowest agreement observed among children aged 1–4 years. Although these findings should be interpreted cautiously, they indicate that challenges in pediatric triage may vary across developmental stages. Visit type analyses also showed that disease encounters had more frequent discordance toward higher prehospital acuity than non-disease encounters in the pediatric cohort. This pattern is consistent with prior prehospital literature suggesting that non-trauma presentations may be more complex to assess and may prompt more conservative transport or acuity decisions when clinical uncertainty is high [31,32]. These findings suggest that less externally apparent or more heterogeneous clinical presentations may contribute to cross-setting disagreement.

Similarly, the exploratory analysis of pediatric encounters assigned prehospital Pre-KTAS level 2 showed that seizure/convulsion and fever accounted for the largest proportion of cases, and discordance toward higher prehospital acuity was more frequent than exact agreement across most complaint categories. These findings may reflect complaint-specific triage challenges, particularly for presentations with potentially serious but variable clinical trajectories, and identify pediatric Pre-KTAS level 2 as a practical target for focused EMS education, case-based feedback, and future refinement of pediatric prehospital triage guidance.

### Practice and system implications

From a practice and system perspective, these results support pediatric-focused Pre-KTAS quality improvement during the early implementation phase. Discordance toward higher prehospital acuity in children may reflect appropriately cautious decision-making in uncertain presentations, but frequent discordance of this direction may also increase resource activation and contribute to ED crowding. Improvement efforts should therefore aim to enhance consistency at key decision points while maintaining patient safety.

In line with prior pediatric triage studies and reviews, training, simulation, and structured feedback for Pre-KTAS should prioritize the 2–3 and 3–4 boundaries and incorporate pediatric-specific scenarios, including age-based vital sign interpretation, non-specific presentations such as fever, seizure, altered responsiveness, and modifier application [28–30,33]. The concentration of discordance among pediatric Pre-KTAS level 2 encounters further suggests that complaint-specific feedback may be useful, particularly for presentations with potentially serious but variable clinical trajectories.

At the system level, routine linkage-based audit comparing EMS provider-assigned Pre-KTAS, initial ED KTAS, and downstream clinical disposition may help distinguish high-risk discordance toward lower prehospital acuity from high-volume discordance toward higher prehospital acuity. Such audit-and-feedback systems may support continuous calibration of the nationwide prehospital triage framework, promote more consistent resource allocation across the prehospital–ED continuum, and provide a foundation for future multicenter evaluation of pediatric triage performance.

### Limitations

Several limitations should be considered. First, selection bias is possible because 453 encounters were excluded for missing Pre-KTAS levels (n = 328) or no completed ED registration after EMS transport (n = 125). Comparisons of available baseline characteristics showed that excluded encounters had a higher proportion of non-disease encounters and, among those with missing Pre-KTAS, a lower proportion of pediatric encounters (S1 Table). These differences should be considered when interpreting the findings.

Second, this was a single-center study conducted at Bundang CHA Medical Center, a university-affiliated hospital that functions as both a regional emergency medical center and a specialized pediatric emergency center. This setting may have influenced the case mix, including the proportion of pediatric emergencies, high-acuity encounters, and interfacility transfers. Triage patterns may also reflect institution-specific protocols, staffing patterns, and provider experience. Therefore, the findings may not be directly generalizable to other hospital types or regions in Korea, and multicenter studies are needed to confirm the external validity of these results.

Third, the dataset did not contain the precise timestamp indicating when Pre-KTAS was assigned within the EMS mission. Although Pre-KTAS was recorded during the prehospital encounter after patient contact and before ED registration, we could not determine whether the recorded level reflected the field assessment, reassessment during transport, or the patient’s status immediately before ED arrival. In addition, the individual EMS provider who assigned the Pre-KTAS level was not recorded, precluding direct analysis of provider-level qualification, experience, or training effects on triage concordance.

Fourth, initial ED KTAS was used as a pragmatic comparator, not as a definitive gold standard. ED triage can be influenced by local operational conditions, including crowding, staffing, triage workload, and practice patterns, and may not fully reflect the prehospital context. Accordingly, discordance should be interpreted as disagreement between settings rather than definitive misclassification by either system. Because the dataset did not include independent adjudication of triage appropriateness or outcome-based validation, this study cannot determine which classification was “correct” at the individual encounter level.

Finally, κ statistics are sensitive to category prevalence and marginal distributions. Therefore, κ values should be interpreted alongside the observed disagreement structure, including the concentration of discordance at adjacent-level boundaries. Sensitivity analyses using Gwet’s AC1 yielded higher agreement estimates than Cohen’s κ in both age groups, but the relative pattern of lower agreement in pediatric encounters was preserved, suggesting that the observed age-group differences were not solely attributable to prevalence-related limitations of κ.

## Conclusion

During the first year of nationwide Pre-KTAS implementation, agreement between EMS provider-assigned prehospital Pre-KTAS and initial ED KTAS was modest, with lower concordance in pediatric encounters than in adult encounters and discordance concentrated mainly at adjacent triage levels. These findings suggest practical targets for pediatric-focused quality improvement, including age-specific vital sign interpretation, modifier application in non-specific presentations, and decision-making at borderline acuity levels. At the system level, linkage-based feedback between EMS agencies and EDs may support continuous calibration of Pre-KTAS, pediatric-focused quality improvement, and more consistent resource allocation across the prehospital–ED continuum.

## Supporting information

S1 Table Comparison of baseline characteristics between included and excluded encounters. Categorical variables are presented as n (%) and compared using Pearson’s chi-square test. Age is presented as median (IQR) and compared using the Wilcoxon rank-sum test. P values compare each excluded group with the included group. ^a^ Encounters excluded for missing prehospital Pre-KTAS level. Sex and visit type comparisons were based on n = 5,033 due to 24 encounters with missing sex information. ^b^ Of the 125 encounters with no completed ED registration after EMS transport, 117 were identified through hospital records, of which 114 could be linked to EMS run-sheet data for comparison using prehospital variables. The remaining 11 encounters (3 unlinked to EMS data + 8 untraceable) could not be compared.
(DOCX)

S2 Table Cross-tabulation of prehospital pre-KTAS and initial ED KTAS by age group. Panel A. Pediatric encounters(<15 years, n = 1,242) [5x5 table]. Panel B. Adult encounters (≥*15 years, n = 3,487)* [5x5 table]. Values are counts (n) of encounters. Rows indicate Pre-KTAS levels assigned by EMS and columns indicate initial ED KTAS levels assigned at ED registration (1 = highest acuity, 5 = lowest acuity). Pediatric encounters were defined as <15 years and adult encounters as ≥15 years according to the KTAS age threshold. Encounters with missing Pre-KTAS or missing ED KTAS were excluded; therefore, row and column totals equal the age-group sample size. Percentages are intentionally not shown; see Fig. 2 for row-normalized proportions.
(DOCX)

S3 Table Sensitivity analysis of agreement using Gwet’s AC1 coefficient alongside kappa statistics. Gwet’s AC1 was computed to assess the robustness of agreement estimates given the known sensitivity of Cohen’s kappa to skewed marginal distributions. When category prevalence is imbalanced, kappa may underestimate agreement (the kappa paradox). AC1 addresses this limitation by using a chance-corrected agreement measure that is less dependent on category prevalence. In both pediatric and adult groups, AC1 values were higher than unweighted kappa, while the overall pattern of lower agreement in pediatric encounters was preserved, suggesting that the findings were not primarily driven by marginal distribution effects. Unweighted kappa values are presented for comparison; full agreement statistics are reported in Table 2. Ninety-five percent confidence intervals were estimated using large-sample standard errors.
(DOCX)

S4 Table Agreement between prehospital pre-KTAS and initial ED KTAS across pediatric age subgroups. Pre-KTAS indicates the Prehospital Korean Triage and Acuity Scale assigned by EMS providers; initial ED KTAS indicates the Korean Triage and Acuity Scale level assigned at ED registration (1 = highest acuity). Unweighted κ was calculated using Cohen’s kappa. Quadratically weighted κ was calculated to account for the ordinal five-level triage scale. Ninety-five percent confidence intervals were estimated using asymptotic standard errors. “Discordance toward higher prehospital acuity” indicates that the Pre-KTAS level was numerically lower than the initial ED KTAS level; “discordance toward lower prehospital acuity” indicates that the Pre-KTAS level was numerically higher than the initial ED KTAS level. These terms describe the direction of discordance only and do not imply clinical correctness or superiority of either triage assignment.
(DOCX)

S5 Table Agreement and discordance direction stratified by visit type and age group. Visit type was classified as disease or non-disease according to the routinely recorded source-data field. The non-disease category included injury-related or external-cause presentations, including trauma, burns, and poisoning, as recorded in the source data. P values compare discordance direction categories (discordance toward higher prehospital acuity, exact agreement, and discordance toward lower prehospital acuity) between disease and non-disease encounters within each age group using the chi-square test.
(DOCX)

S6 Table Chief complaint distribution and direction of discordance among pediatric encounters assigned prehospital pre-KTAS level 2. Chief complaints were extracted from EMS run-sheet free-text fields and categorized by keyword matching. Higher prehospital acuity indicates that the Pre-KTAS level was numerically lower (more urgent) than the initial ED KTAS level, whereas lower prehospital acuity indicates that the Pre-KTAS level was numerically higher (less urgent) than the initial ED KTAS level. Agreement rate denotes the proportion of encounters with exact concordance between prehospital Pre-KTAS and initial ED KTAS within each chief complaint category. Row percentages for agreement, higher prehospital acuity, and lower prehospital acuity sum to 100% within each chief complaint category. †Other includes allergic reaction (n = 1), gastrointestinal complaints (n = 1), and unclassified complaints (n = 30).
(DOCX)

S7 Table EMS crew composition and exploratory analysis of agreement between prehospital pre-KTAS and initial ED KTAS. EMS crew composition and exploratory agreement analysis. Panel A describes the distribution of EMS crew composition, including the presence of a nurse, EMT Level 1 without a nurse, EMT Level 2/other only, and crew size. Panel B presents exploratory agreement between prehospital Pre-KTAS and initial ED KTAS stratified by crew composition for pediatric (<15 years) and adult (≥15 years) encounters. Overall agreement and kappa statistics (unweighted and quadratically weighted) are reported. Crew composition was used as a proxy for provider training level, as individual-level qualification data were not available. These analyses are exploratory and should be interpreted with caution. *The EMT Level 2/other only group was very small and is presented descriptively only.
(DOCX)
