# Supplementary material for: Agreement between EMS provider-assigned prehospital triage and initial emergency department triage in pediatric and adult EMS-transported encounters: A retrospective observational study
Source: PLoS One. 2026 Jul 6;21(7):e0352969. doi: 10.1371/journal.pone.0352969 (PMC13336163; doi:10.1371/journal.pone.0352969)
Supplement: S1 Table — Categorical variables are presented as n (%) and compared using Pearson’s chi-square test. Age is presented as median (IQR) and compared using the Wilcoxon rank-sum test. P values compare each excluded group with the included group. a Encounters excluded for missing prehospital Pre-KTAS level. Sex and visit type comparisons were based on n = 5,033 due to 24 encounters with missing sex information. b Of the 125 encounters with no completed ED registration after EMS transport, 117 were identified through hospital records, of which 114 could be linked to EMS run-sheet data for comparison using prehospital variables. The remaining 11 encounters (3 unlinked to EMS data + 8 untraceable) could not be compared. (DOCX) [file pone.0352969.s001.docx]

**S1 Table. Comparison of baseline characteristics between included and excluded encounters.**

|  | **Analyzed cohort** | **Missing Pre-KTAS** | **P Value** | **Cancelled ED registration** | **P Value** |
| --- | --- | --- | --- | --- | --- |
|  | (n = 4,729) | (n = 328)^a^ |  | (n = 114)^b^ |  |
| Male, n (%) | 2,659 (56.2) | 157 (51.6) | 0.119 | 54 (47.4) | 0.060 |
| Visit type |  |  | <0.001 |  | <0.001 |
| Disease, n (%) | 4,104 (86.8) | 239 (78.6) |  | 86 (75.4) |  |
| Non-disease, n (%) | 580 (12.3) | 63 (20.7) |  | 18 (15.8) |  |
| Others, n (%) | 45 (1.0) | 2 (0.7) |  | 10 (8.8) |  |
| Pediatric (<15 years), n (%) | 1,242 (26.3) | 46 (15.1) | <0.001 | 24 (21.1) | 0.211 |
| Age, years, median (IQR) | 60 (12–75) | 61 (34–76.5) | 0.018 | 44.5 (29–67) | 0.011 |

*Categorical variables are presented as n (%) and compared using Pearson’s chi-square test. Age is presented as median (IQR) and compared using the Wilcoxon rank-sum test. P values compare each excluded group with the included group.*

*^a^ Encounters excluded for missing prehospital Pre-KTAS level. Sex and visit type comparisons were based on n = 5,033 due to 24 encounters with missing sex information.*

*^b^ Of the 125 encounters with no completed ED registration after EMS transport, 117 were identified through hospital records, of which 114 could be linked to EMS run-sheet data for comparison using prehospital variables. The remaining 11 encounters (3 unlinked to EMS data + 8 untraceable) could not be compared.*
