# Supplementary material for: Agreement between EMS provider-assigned prehospital triage and initial emergency department triage in pediatric and adult EMS-transported encounters: A retrospective observational study
Source: PLoS One. 2026 Jul 6;21(7):e0352969. doi: 10.1371/journal.pone.0352969 (PMC13336163; doi:10.1371/journal.pone.0352969)
Supplement: S2 Table — Values are counts (n) of encounters. Rows indicate Pre-KTAS levels assigned by EMS and columns indicate initial ED KTAS levels assigned at ED registration (1 = highest acuity, 5 = lowest acuity). Pediatric encounters were defined as <15 years and adult encounters as ≥15 years according to the KTAS age threshold. Encounters with missing Pre-KTAS or missing ED KTAS were excluded; therefore, row and column totals equal the age-group sample size. Percentages are intentionally not shown; see Fig. 2 for row-normalized proportions. (DOCX) [file pone.0352969.s002.docx]

**S2 Table. Cross-tabulation of prehospital Pre-KTAS and initial ED KTAS by age group.**

**Panel A. Pediatric encounters(<15 years, n = 1,242) [5x5 table]**

|  | **ED-KTAS level** | | | | |  |
| --- | --- | --- | --- | --- | --- | --- |
|  | **1** | **2** | **3** | **4** | **5** | **Total** |
| **Pre-KTAS level** |  |  |  |  |  |  |
| **1** | 18 | 5 | 12 | 1 | 0 | 36 |
| **2** | 9 | 33 | 246 | 26 | 3 | 317 |
| **3** | 3 | 24 | 400 | 145 | 13 | 585 |
| **4** | 0 | 18 | 94 | 137 | 19 | 268 |
| **5** | 0 | 4 | 10 | 18 | 4 | 36 |
| **Total** | 30 | 84 | 762 | 327 | 39 | 1242 |

**Panel B . Adult encounters (≥15 years, n = 3,487) [5x5 table]**

|  | **ED-KTAS level** | | | | |  |
| --- | --- | --- | --- | --- | --- | --- |
|  | **1** | **2** | **3** | **4** | **5** | **Total** |
| **Pre-KTAS level** |  |  |  |  |  |  |
| **1** | 238 | 42 | 18 | 2 | 0 | 300 |
| **2** | 190 | 591 | 446 | 32 | 5 | 1264 |
| **3** | 56 | 314 | 922 | 106 | 13 | 1411 |
| **4** | 14 | 82 | 258 | 82 | 20 | 456 |
| **5** | 3 | 9 | 28 | 7 | 9 | 56 |
| **Total** | 501 | 1038 | 1672 | 229 | 47 | 3487 |

*Values are counts (n) of encounters. Rows indicate Pre-KTAS levels assigned by EMS and columns indicate initial ED KTAS levels assigned at ED registration (1=highest acuity, 5=lowest acuity). Pediatric encounters were defined as <15 years and adult encounters as ≥15 years according to the KTAS age threshold. Encounters with missing Pre-KTAS or missing ED KTAS were excluded; therefore, row and column totals equal the age-group sample size. Percentages are intentionally not shown; see Fig. 2 for row-normalized proportions.*
