# Supplementary material for: Agreement between EMS provider-assigned prehospital triage and initial emergency department triage in pediatric and adult EMS-transported encounters: A retrospective observational study
Source: PLoS One. 2026 Jul 6;21(7):e0352969. doi: 10.1371/journal.pone.0352969 (PMC13336163; doi:10.1371/journal.pone.0352969)
Supplement: S3 Table — Gwet’s AC1 was computed to assess the robustness of agreement estimates given the known sensitivity of Cohen’s kappa to skewed marginal distributions. When category prevalence is imbalanced, kappa may underestimate agreement (the kappa paradox). AC1 addresses this limitation by using a chance-corrected agreement measure that is less dependent on category prevalence. In both pediatric and adult groups, AC1 values were higher than unweighted kappa, while the overall pattern of lower agreement in pediatric encounters was preserved, suggesting that the findings were not primarily driven by marginal distribution effects. Unweighted kappa values are presented for comparison; full agreement statistics are reported in Table 2. Ninety-five percent confidence intervals were estimated using large-sample standard errors. (DOCX) [file pone.0352969.s003.docx]

**S3 Table. Sensitivity analysis of agreement using Gwet’s AC1 coefficient alongside kappa statistics.**

| **Group** | **Encounters (n)** | **Unweighted κ (95% CI)** | **Gwet's AC1 (95% CI)** |
| --- | --- | --- | --- |
| Pediatric (<15 years) | 1,242 | 0.18 (0.14–0.22) | 0.38 (0.33–0.43) |
| Adult (≥15 years) | 3,487 | 0.30 (0.28–0.33) | 0.43 (0.40–0.46) |

*Gwet’s AC1 was computed to assess the robustness of agreement estimates given the known sensitivity of Cohen’s kappa to skewed marginal distributions. When category prevalence is imbalanced, kappa may underestimate agreement (the kappa paradox). AC1 addresses this limitation by using a chance-corrected agreement measure that is less dependent on category prevalence. In both pediatric and adult groups, AC1 values were higher than unweighted kappa, while the overall pattern of lower agreement in pediatric encounters was preserved, suggesting that the findings were not primarily driven by marginal distribution effects. Unweighted kappa values are presented for comparison; full agreement statistics are reported in Table 2. Ninety-five percent confidence intervals were estimated using large-sample standard errors.*
