# Supplementary material for: Agreement between EMS provider-assigned prehospital triage and initial emergency department triage in pediatric and adult EMS-transported encounters: A retrospective observational study
Source: PLoS One. 2026 Jul 6;21(7):e0352969. doi: 10.1371/journal.pone.0352969 (PMC13336163; doi:10.1371/journal.pone.0352969)
Supplement: S4 Table — Pre-KTAS indicates the Prehospital Korean Triage and Acuity Scale assigned by EMS providers; initial ED KTAS indicates the Korean Triage and Acuity Scale level assigned at ED registration (1 = highest acuity). Unweighted κ was calculated using Cohen’s kappa. Quadratically weighted κ was calculated to account for the ordinal five-level triage scale. Ninety-five percent confidence intervals were estimated using asymptotic standard errors. “Discordance toward higher prehospital acuity” indicates that the Pre-KTAS level was numerically lower than the initial ED KTAS level; “discordance toward lower prehospital acuity” indicates that the Pre-KTAS level was numerically higher than the initial ED KTAS level. These terms describe the direction of discordance only and do not imply clinical correctness or superiority of either triage assignment. (DOCX) [file pone.0352969.s004.docx]

**S4 Table. Agreement between prehospital Pre-KTAS and initial ED KTAS across pediatric age subgroups.**

|  | **<1 year (n = 228)** | **1–4 years (n = 681)** | **5–14 years (n = 333)** | **All pediatric (n = 1,242)** |
| --- | --- | --- | --- | --- |
| **Overall agreement,** % | 50.9 | 46 | 49 | 47.7 |
| **Unweighted κ** (95% CI) | 0.29 (0.20–0.38) | 0.12 (0.07–0.17) | 0.19 (0.11–0.27) | 0.18 (0.14–0.22) |
| **Quadratically weighted κ** (95% CI) | 0.41 (0.32–0.49) | 0.23 (0.18–0.28) | 0.24 (0.16–0.33) | 0.27 (0.23–0.31) |
| **Discordance direction** |  |  |  |  |
| Higher prehospital acuity, n (%) | 71 (31.1) | 279 (41.0) | 120 (36.0) | 470 (37.8) |
| Agreement, n (%) | 116 (50.9) | 313 (46.0) | 163 (49.0) | 592 (47.7) |
| Lower prehospital acuity, n (%) | 41 (18.0) | 89 (13.1) | 50 (15.0) | 180 (14.5) |

*Pre-KTAS indicates the Prehospital Korean Triage and Acuity Scale assigned by EMS providers; initial ED KTAS indicates the Korean Triage and Acuity Scale level assigned at ED registration (1 = highest acuity). Unweighted κ was calculated using Cohen’s kappa. Quadratically weighted κ was calculated to account for the ordinal five-level triage scale. Ninety-five percent confidence intervals were estimated using asymptotic standard errors. “Discordance toward higher prehospital acuity” indicates that the Pre-KTAS level was numerically lower than the initial ED KTAS level; “discordance toward lower prehospital acuity” indicates that the Pre-KTAS level was numerically higher than the initial ED KTAS level. These terms describe the direction of discordance only and do not imply clinical correctness or superiority of either triage assignment.*
