# Supplementary material for: Agreement between EMS provider-assigned prehospital triage and initial emergency department triage in pediatric and adult EMS-transported encounters: A retrospective observational study
Source: PLoS One. 2026 Jul 6;21(7):e0352969. doi: 10.1371/journal.pone.0352969 (PMC13336163; doi:10.1371/journal.pone.0352969)
Supplement: S5 Table — Visit type was classified as disease or non-disease according to the routinely recorded source-data field. The non-disease category included injury-related or external-cause presentations, including trauma, burns, and poisoning, as recorded in the source data. P values compare discordance direction categories (discordance toward higher prehospital acuity, exact agreement, and discordance toward lower prehospital acuity) between disease and non-disease encounters within each age group using the chi-square test. (DOCX) [file pone.0352969.s005.docx]

**S5 Table. Agreement and discordance direction stratified by visit type and age group.**

| **Group** | **n** | **Exact agreement, %** | **Discordance toward higher prehospital acuity, %** | **Discordance toward lower prehospital acuity, %** | **Unweighted κ (95% CI)** | **Weighted κ (95% CI)** |
| --- | --- | --- | --- | --- | --- | --- |
| **Pediatric** |  |  |  |  |  |  |
| Disease | 1,016 | 47.2 | 39.3 | 13.5 | 0.13 (0.08–0.17) | 0.22 (0.18–0.27) |
| Non-disease | 222 | 49.1 | 31.5 | 19.4 | 0.14 (0.04–0.24) | 0.20 (0.09–0.32) |
| **Adult** |  |  |  |  |  |  |
| Disease | 3,088 | 53.3 | 18.7 | 28 | 0.29 (0.27–0.32) | 0.38 (0.35–0.40) |
| Non-disease | 358 | 48.9 | 26 | 25.1 | 0.32 (0.25–0.39) | 0.48 (0.42–0.55) |

*Visit type was classified as disease or non-disease according to the routinely recorded source-data field. The non-disease category included injury-related or external-cause presentations, including trauma, burns, and poisoning, as recorded in the source data. P values compare discordance direction categories (discordance toward higher prehospital acuity, exact agreement, and discordance toward lower prehospital acuity) between disease and non-disease encounters within each age group using the chi-square test.*
