# Supplementary material for: Agreement between EMS provider-assigned prehospital triage and initial emergency department triage in pediatric and adult EMS-transported encounters: A retrospective observational study
Source: PLoS One. 2026 Jul 6;21(7):e0352969. doi: 10.1371/journal.pone.0352969 (PMC13336163; doi:10.1371/journal.pone.0352969)
Supplement: S6 Table — Chief complaints were extracted from EMS run-sheet free-text fields and categorized by keyword matching. Higher prehospital acuity indicates that the Pre-KTAS level was numerically lower (more urgent) than the initial ED KTAS level, whereas lower prehospital acuity indicates that the Pre-KTAS level was numerically higher (less urgent) than the initial ED KTAS level. Agreement rate denotes the proportion of encounters with exact concordance between prehospital Pre-KTAS and initial ED KTAS within each chief complaint category. Row percentages for agreement, higher prehospital acuity, and lower prehospital acuity sum to 100% within each chief complaint category. †Other includes allergic reaction (n = 1), gastrointestinal complaints (n = 1), and unclassified complaints (n = 30). (DOCX) [file pone.0352969.s006.docx]

**S6 Table. Chief complaint distribution and direction of discordance among pediatric encounters assigned prehospital Pre-KTAS level 2.**

| **Chief complaint** | **n (%)** | **Agreement, n (%)** | **Higher prehospital acuity, n (%)** | **Lower prehospital acuity, n (%)** |
| --- | --- | --- | --- | --- |
| Seizure/convulsion | 187 (59.0) | 11 (5.9) | 170 (90.9) | 6 (3.2) |
| Fever | 62 (19.6) | 9 (14.5) | 52 (83.9) | 1 (1.6) |
| Respiratory distress | 18 (5.7) | 5 (27.8) | 12 (66.7) | 1 (5.6) |
| Abdominal pain | 9 (2.8) | 0 (0.0) | 9 (100.0) | 0 (0.0) |
| Altered mental status/syncope | 5 (1.6) | 1 (20.0) | 3 (60.0) | 1 (20.0) |
| Vomiting | 4 (1.3) | 2 (50.0) | 2 (50.0) | 0 (0.0) |
| Other | 32 (10.1)^†^ | 5 (15.6) | 27 (84.4) | 0 (0.0) |
| **Total** | **317 (100)** | **33 (10.4)** | **275 (86.8)** | **9 (2.8)** |

*Chief complaints were extracted from EMS run-sheet free-text fields and categorized by keyword matching. Higher prehospital acuity indicates that the Pre-KTAS level was numerically lower (more urgent) than the initial ED KTAS level, whereas lower prehospital acuity indicates that the Pre-KTAS level was numerically higher (less urgent) than the initial ED KTAS level. Agreement rate denotes the proportion of encounters with exact concordance between prehospital Pre-KTAS and initial ED KTAS within each chief complaint category. Row percentages for agreement, higher prehospital acuity, and lower prehospital acuity sum to 100% within each chief complaint category. †Other includes allergic reaction (n = 1), gastrointestinal complaints (n = 1), and unclassified complaints (n = 30).*
