# Supplementary material for: Agreement between EMS provider-assigned prehospital triage and initial emergency department triage in pediatric and adult EMS-transported encounters: A retrospective observational study
Source: PLoS One. 2026 Jul 6;21(7):e0352969. doi: 10.1371/journal.pone.0352969 (PMC13336163; doi:10.1371/journal.pone.0352969)
Supplement: S7 Table — EMS crew composition and exploratory agreement analysis. Panel A describes the distribution of EMS crew composition, including the presence of a nurse, EMT Level 1 without a nurse, EMT Level 2/other only, and crew size. Panel B presents exploratory agreement between prehospital Pre-KTAS and initial ED KTAS stratified by crew composition for pediatric (<15 years) and adult (≥15 years) encounters. Overall agreement and kappa statistics (unweighted and quadratically weighted) are reported. Crew composition was used as a proxy for provider training level, as individual-level qualification data were not available. These analyses are exploratory and should be interpreted with caution. *The EMT Level 2/other only group was very small and is presented descriptively only. (DOCX) [file pone.0352969.s007.docx]

**S7 Table. EMS crew composition and exploratory analysis of agreement between prehospital Pre-KTAS and initial ED KTAS.**

**Panel A. Crew qualification composition**

|  | **All (n = 4,729)** | **Pediatric (n = 1,242)** | **Adult (n = 3,487)** |
| --- | --- | --- | --- |
| **Crew qualification group** |  |  |  |
| Nurse in crew | 3,119 (66.0) | 862 (69.4) | 2,257 (64.7) |
| EMT Level 1, no nurse | 1,602 (33.9) | 378 (30.4) | 1,224 (35.1) |
| EMT Level 2/other only | 8 (0.2)* | 2 (0.2)* | 6 (0.2)* |
| **Crew size** |  |  |  |
| 2 members | 2,634 (55.7) | 715 (57.6) | 1,919 (55.0) |
| 3 members | 2,089 (44.2) | 526 (42.3) | 1,563 (44.8) |

**Panel B. Exploratory agreement by crew qualification group**

|  | **Nurse in crew** | **EMT Level 1, no nurse** |
| --- | --- | --- |
| **Pediatric (<15 years)** |  |  |
| Encounters, n | 862 | 378 |
| Overall agreement, % | 47.2 | 48.1 |
| Unweighted κ (95% CI) | 0.18 (0.13–0.23) | 0.17 (0.10–0.24) |
| Quadratically weighted κ (95% CI) | 0.27 (0.22–0.32) | 0.28 (0.21–0.35) |
| **Adult (≥15 years)** |  |  |
| Encounters, n | 2,257 | 1,224 |
| Overall agreement, % | 51.5 | 55 |
| Unweighted κ (95% CI) | 0.29 (0.26–0.32) | 0.33 (0.29–0.37) |
| Quadratically weighted κ (95% CI) | 0.39 (0.36–0.42) | 0.43 (0.39–0.47) |

*EMS crew composition and exploratory agreement analysis. Panel A describes the distribution of EMS crew composition, including the presence of a nurse, EMT Level 1 without a nurse, EMT Level 2/other only, and crew size. Panel B presents exploratory agreement between prehospital Pre-KTAS and initial ED KTAS stratified by crew composition for pediatric (<15 years) and adult (≥15 years) encounters. Overall agreement and kappa statistics (unweighted and quadratically weighted) are reported. Crew composition was used as a proxy for provider training level, as individual-level qualification data were not available. These analyses are exploratory and should be interpreted with caution. *The EMT Level 2/other only group was very small and is presented descriptively only.*
